# Rotavirus vaccination is not associated with incident celiac disease or autoimmune thyroid disease in a national cohort of privately insured children

**DOI:** 10.1038/s41598-022-17187-y

**Published:** 2022-07-28

**Authors:** Catherine Kim, Zhe Yin, Neil Kamdar, Grace J. Lee

**Affiliations:** 1 Departments of Medicine, Obstetrics & Gynecology and Epidemiology, University of Michigan, 2800 Plymouth Road, Bldng 16, Room 405E, Ann Arbor, MI 48109-2800 USA; 2 Institute for Health Policy and Innovation, University of Michigan, Ann Arbor, MI 48109 USA; 3 Department of Pediatrics, University of Michigan, Ann Arbor, MI 48109 USA

**Keywords:** Epidemiology, Paediatric research

## Abstract

Rotavirus infection is a potential trigger for autoimmune diseases, and previous reports note associations between rotavirus vaccination and type 1 diabetes. In this report, we examine the association between rotavirus vaccination and autoimmune diseases associated with type 1 diabetes: celiac disease and autoimmune thyroiditis. We conducted a retrospective cohort study using de-identified claims data (Optum Clinformatics^®^ Data Mart). Eligible infants were born between 2001 and 2018 and continuously enrolled from birth for at least 365 days (n = 2,109,225). Twenty-nine percent (n = 613,295) of infants were born prior to the introduction of rotavirus vaccine in 2006; 32% (n = 684,214) were eligible for the vaccine but were not vaccinated; 9.6% (n = 202,016) received partial vaccination, and 28.9% received full vaccination (n = 609,700). There were 1379 cases of celiac disease and 1000 cases of autoimmune thyroiditis. Children who were born prior to the introduction of rotavirus vaccine in 2006 had lower risk of celiac disease compared to unvaccinated children born after 2006 (hazard ratio [HR] 0.71, 95% confidence interval [CI] 0.59, 0.85). However, children who were partially vaccinated (HR 0.90, 95% CI 0.73, 1.11) or fully vaccinated (HR 1.03, 95% CI 0.88, 1.21) had similar risk to eligible, unvaccinated children. Risk of autoimmune thyroiditis was similar by vaccination status. We conclude that rotavirus vaccination is not associated with increased or decreased risk for celiac disease or autoimmune thyroiditis.

## Introduction

Celiac disease (CD) and autoimmune thyroid disease (AT) are common autoimmune conditions of childhood, each estimated to affect up to 1% of children^1–7^. Approximately 10% of individuals with CD also have AT^8^. CD and AT share genetic risk factors for susceptibility, particularly human leukocyte antigen (HLA) alleles DR3-DQ2 which are involved in the antigen presentation process and autoimmunity^9–11^.

Infections may induce autoimmunity in vulnerable individuals through the mechanisms of molecular mimicry, bystander activation, epitope spreading, and presentation of cryptic antigens^11^. Molecular mimicry posits that immunity is initially activated in response to a pathogen during infection, and autoimmunity arises due to cross-reactivity between viral sequences and self-components^12^. The resulting tissue damage releases additional antigens and leads to activation of antigen presenting cells, further exacerbating the autoimmune process. In the bystander activation model, activation of self-reactive T-helper cells is part of a non-specific response to infection, where initial inflammation leads to destruction of tissue, further release of tissue antigen, followed by activation of self-reactive T cells through a T cell receptor dependent mechanism^13^. Epitope spreading models are a variant of this model; in these models, immune responses to chronic infections cause tissue damage and release of self-antigens^14^. Finally, cryptic antigen models propose that self-antigens not usually presented to the immune system are released in response to inflammation, and subsequently lead to alteration of the autoimmune response^14,15^.

CD is triggered by gluten ingestion in wheat, barley, and rye in genetically predisposed individuals^16^. Ingestion of greater quantities of gluten and inflammation from enterovirus infections have been reported to increase local interferon production and expression of tissue transglutaminase^17^. Previous studies have also hypothesized that rotavirus infection, another common cause of viral gastroenteritis, may be a trigger for CD^18,19^.

Thus, it is possible that vaccination against rotavirus (RV) could reduce the tissue damage that is key to bystander activation, epitope spreading model, and cryptic antigen model. Conversely, since rotavirus vaccine is a live vaccine, RV could potentially initiate cross-reactivity and trigger autoimmunity via molecular mimicry. Although rotavirus infection in infancy is believed to infect most children by the age of 5 years^20^, vaccination is typically administered by eight months of age, which could mean exposure at a younger and possibly more vulnerable age than children would otherwise experience. However, previous studies conflict regarding whether RV reduces risk of CD^22–24^. No reports examine whether RV reduces risk of CD in the United States, which was one of the first countries to introduce the vaccine in 2006. Two types of the RV are routinely used in the United States: pentavalent RotaTeq introduced in 2006 and given in 3 doses at 2, 4 and 6 months; and monovalent Rotarix introduced in 2008 and given in 2 doses at 2 months and 4 months^25^.

Rotavirus as a potential trigger for thyroiditis has not been as well studied as type 1 diabetes and celiac disease, although components of several viruses including hepatitis C, human parvovirus B19, coxsackie virus, and herpes virus have been detected in the thyroid of Hashimoto’s thyroiditis patients^26^. However, among mice infected with reovirus type 1 (rotavirus is in the reovirus family) develop thyroiditis^27^, and exposure to reovirus type 1 induced anti-thyroglobulin antibodies^28^. No reports examine whether RV reduces risk of AT, despite shared susceptibility in HLA genes with CD and type 1 diabetes. Since nationwide data regarding dates, types, and doses of vaccines are available from insurance claims data, we conducted a nationwide study to investigate the hypothesis that the RV may reduce the likelihood of these CD and AT. This report is the largest study of RV and CD to date and the only report examining both RV and AT.

## Results

**Figure 1:**
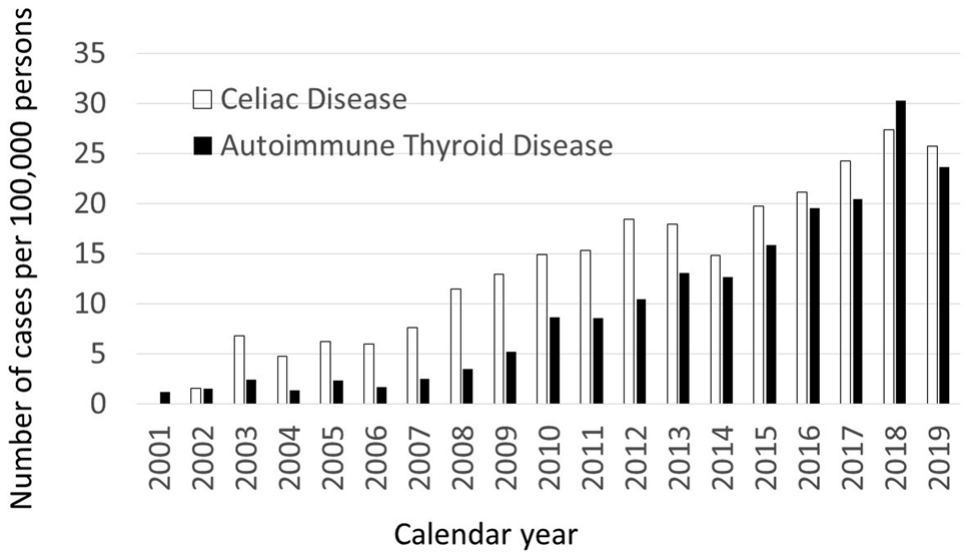
Prevalence of celiac disease and autoimmune thyroid disease in the cohort per 100,000 persons enrolled, by calendar year.

In this cohort of children enrolled from birth between 2001 and 2018, the prevalence of CD and AT cases increased with calendar year. Figure 1 shows the number of cases for a particular calendar year divided by the number of children in the cohort for that particular calendar year. Table 1 shows the characteristics of the study sample by RV status. Children who were eligible for RV, (i.e. born after 2006) but who were not vaccinated were less likely to have attended a well-child visit before the age of 2 years compared to infants born before 2006, partially vaccinated infants, or fully vaccinated infants. Unvaccinated but eligible children had a similar number of days of enrollment compared to partially vaccinated or fully vaccinated children, although fewer days of enrollment compared to children born in earlier years (2001–2005).

**Table 1:** Characteristics by rotavirus vaccination (RV) status (n = 2,109,225).

	Born 2001–2005, prior to RV availability	No RV, born after 2006	Partial RV, born after 2006	Complete RV, born after 2006
	N = 613,295	N = 684,214	N = 202,016	N = 609,700
Well-child visit before age of 2 years (n, %)	511,471 (83.4)	423,859 (62.0)	201,610 (99.8)	609,364 (99.95)
Days of enrollment (person-days at risk) (mean, SD)	1454.05 (1331.4)	1092.28 (966.0)	1461.33 (949.4)	1537.01 (964.0)
Q1 of enrollment, days	360–588	360–456	360–755	360–820
Q2 of enrollment, days	588–963	456–727	755–1125	820–1221
Q3 of enrollment, days	963–1753	727–1345	1125–1828	1221–1971
Q4 of enrollment, days	1753–7302	1345–5478	1828–5478	1971–5463
Female (n, %)	298,250 (48.6)	333,606 (48.8)	98,222 (48.6)	297,673 (48.8)
**Geographic region**				
0 (New England)	39,519 (6.5)	42,840 (6.3)	11,896 (5.9)	30,863 (5.1)
1 (New York, Pennsylvania)	29,960 (4.9)	37,350 (5.5)	10,596 (5.3)	31,687 (5.2)
2 (Mid-Atlantic)	57,997 (9.5)	62,839 (9.2)	18,144 (9.0)	57,629 (9.5)
3 (Southeast)	98,074 (16.0)	101,637 (14.9)	29,984 (14.8)	91,336 (15.0)
4 (Midwest)	75,675 (12.3)	59,963 (8.8)	16,353 (8.1)	50,986 (8.4)
5 (North Central)	61,687 (10.1)	61,327 (9.0)	17,630 (8.7)	65,752 (10.8)
6 (Mid Central)	61,701 (10.1)	58,239 (8.5)	16,620 (8.2)	58,703 (9.6)
7 (South Central)	85,629 (14.0)	108,498 (15.9)	32,883 (16.3)	98,465 (16.2)
8 (Mountain)	57,154 (9.3)	70,497 (10.3)	20,103 (10.0)	58,333 (9.6)
9 (Pacific)	45,739 (7.5)	80,920 (11.8)	27,794 (13.8)	65,900 (10.8)
Seizure history (n, %)	19,395 (3.2)	13,967 (2.0)	6600 (3.3)	18,889 (3.1)
Vaccine allergy (n, %)	3085 (0.5)	2707 (0.4)	1284 (0.6)	4256 (0.7)
Preterm birth (n, %)	33,121 (5.4)	20,442 (3.0)	13,219 (6.5)	52,527 (8.6)
Modified childhood chronic conditions score (mean, SD)	0.42 (1.43)	0.25 (1.18)	0.44 (1.52)	0.44 (1.45)
Older sibling with CD (n, %)	133 (0.02)	191 (0.03)	78 (0.04)	361 (0.06)
Older sibling with AT (n, %)	98 (0.02)	83 (0.01)	35 (0.02)	188 (0.03)
Older sibling with autism (n, %)	1074 (0.18)	1973 (0.29)	943 (0.47)	3472 (0.57)

N (%) or mean (SD) shown.

Unvaccinated but vaccine-eligible children were also slightly less likely to report histories of seizures, vaccine allergies, or pre-term birth than other groups of children. Unvaccinated children born after 2006 also had a lower burden of chronic conditions, older siblings with CD, AT, or autism.

Table 2 shows the associations between RV status and risk of CD. Compared to children who were born after the introduction of RV and were not vaccinated, children born before the introduction of RV had a lower risk of CD. These associations persisted after adjustment for multiple factors, including year of birth, childhood health status as represented by comorbidity score and preterm birth, older siblings with CD or autism, and children who had at least one well-child visit. However, partial vaccination and complete vaccination were not associated with risk of CD.

**Table 2:** Risk of celiac disease by rotavirus vaccination (RV) status.

	Hazard ratio (95% confidence interval)	p-value
**Model 1, Adjusted for sex, year of birth, geographic region, vaccine allergy, history of seizures, and attendance of well-child visit**		
No RV, born prior to introduction of RV	0.71 (0.59, 0.85)	0.0002
No RV, born after introduction of RV	Reference	
Partial RV	0.90 (0.73, 1.11)	0.33
Complete RV	1.03 (0.88, 1.21)	0.71
**Model 2, Adjusted for factors in Model 1, and comorbid illness (preterm birth, and childhood comorbidity score)**		
No RV, born prior to introduction of RV	0.69 (0.58, 0.83)	< 0.0001
No RV, born after introduction of RV	Reference	
Partial RV	0.90 (0.72, 1.11)	0.31
Complete RV	1.03 (0.88, 1.20)	0.74
**Model 3, Adjusted for factors in Model 1, and older sibling celiac disease**		
No RV, born prior to introduction of RV	0.70 (0.59, 0.85)	0.0002
No RV, born after introduction of RV	Reference	
Partial RV	0.90 (0.72, 1.11)	0.31
Complete RV	1.01 (0.86, 1.18)	0.95

Other than birth prior to the introduction of RV in 2006, several other factors were associated with risk of CD (Ancillary Table 1). In the base model (Model 1), female sex (hazard ratio [HR] 1.68, 95% confidence interval [CI] 1.51, 1.87), history of seizure (HR 1.41, 95% CI 1.10, 1.81), and allergy to vaccination (HR 1.80, 95% CI 1.14, 2.83) were all associated with an increased risk of CD, as was attendance of a well-child visit (HR 54.8, 95% CI 7.74, 387.7). Geographic regions are defined via United States Postal Service zipcodes, where the first digit of a USA ZIP code generally represents a group of states (https://www.unitedstateszipcodes.org/). Compared to residence in Pacific states (region 9), residence in New England (region 0) (HR 1.95, 95% CI 1.53, 2.49) and New York or Pennsylvania (region 1) (HR 2.00, 95% CI 1.55, 2.58) were associated with greater risk of CD, whereas residence in the southeast (region 3) (95% CI 0.70, 95% CI 0.55, 0.88) was associated with lower risk, consistent with previous reports of greater disease risk in northern latitudes. Residence in the south-central states (HR 0.77, 95% CI 0.61, 0.97) or mountain states (region 8) (HR 1.51, 95% CI 1.21, 1.88) was also associated with greater risk of CD. However, year of birth was not associated with increased risk of CD (HR 0.98, 95% CI 0.96, 1.01). In Model 2, which examined the association between childhood conditions and CD, higher comorbidity burden was associated with greater risk of CD (HR 1.28, 95% CI 1.26, 1.30) but preterm birth was not. Sibling history of CD was strongly associated with risk of CD in the index child (HR 68.30, 95% CI 53.2, 87.7), as was sibling history of autism (HR 1.88, 95% CI 1.11, 3.19).

Table 3 shows the associations between RV status and risk of AT. Compared to children who were born after the introduction of RV and were not vaccinated, children born before the introduction of RV had similar risk of AT. The lack of association persisted after adjustment for multiple factors including year of birth, childhood health status, older siblings with AT or autism, and children who had at least one well-child visit. Partial vaccination and complete vaccination were also not associated with decreased risk of AT.

**Table 3:** Risk of autoimmune thyroid disease by rotavirus vaccination (RV) status.

	Hazard ratio (95% confidence interval)	p-value
**Model 1, Adjusted for sex, year of birth, geographic region, vaccine allergy, history of seizures, and attendance of well-child visit**		
No RV, born prior to introduction of RV	0.81 (0.59, 1.11)	0.19
No RV, born after introduction of RV	Reference	
Partial RV	0.90 (0.63, 1.30)	0.59
Complete RV	0.984 (0.75, 1.29)	0.91
**Model 2, Adjusted for factors in Model 1, preterm birth, and childhood comorbidity score**		
No RV, born prior to introduction of RV	0.79 (0.58, 1.08)	0.14
No RV, born after introduction of RV	Reference	
Partial RV	0.90 (0.62, 1.29)	0.56
Complete RV	0.99 (0.75, 1.30)	0.95
**Model 3, Adjusted for factors in Model 1, older siblings with AT**		
No RV, born prior to introduction of RV	0.82 (0.60, 1.12)	0.22
No RV, born after introduction of RV	Reference	
Partial RV	0.90 (0.63, 1.30)	0.58
Complete RV	0.98 (0.74, 1.28)	0.85

Several other factors were associated with risk of AT (Ancillary Table 2). Model 1, female sex (hazard ratio [HR] 2.31, 95% confidence interval [CI] 1.91, 2.80), history of seizure (HR 2.80, 95% CI 2.06, 3.81), were all associated with an increased risk of AT, as was attendance of a well-child visit. Compared to residence in Pacific states, residence in New York or Pennsylvania (HR 2.49, 95% CI 1.65, 3.74) was associated with greater risk of AT, whereas residence in the North Central (HR 0.63, 95% CI 0.40, 0.98) and mountain states (HR 0.58, 95% CI 0.36, 0.93) was associated with a lower risk of AT. However, year of birth was not associated with increased risk of AT (HR 1.02, 95% CI 0.97, 1.06). In Model 2, which examined the association between childhood conditions and AT, higher chronic comorbidity index score was associated with greater risk of AT (HR 1.39, 95% CI 1.35, 1.42), but preterm birth was not. Sibling history of AT was strongly associated with risk in the index child (HR 38.7, 95% CI 21.8, 68.9).

In sensitivity analyses, we found a similar pattern of results when we excluded children who had not attended a well-child visit (Ancillary Table 3) and when we excluded children who were worn prematurely (Ancillary Table 3). The interaction term for type of rotavirus vaccine (Rotarix vs. Rotateq) was not significant for either CD (p = 0.69) or AT (p = 0.57), suggesting that these vaccines did not differ from each other respect to disease risk. When we examined children who were enrolled until at least the age of 5 years, we found a similar pattern of associations except the lower CD risk of children born prior to 2006 was attenuated (Ancillary Table 4).

## Discussion

Using a large national insurance database from the United States, we found that the prevalence of both CD and AT have increased over time. When we examined whether RV was associated with risk for diseases with overlapping HLA risk alleles, we did not find an association between RV and CD or between RV and AT in unadjusted or adjusted analyses. This suggests that in a broad population (as opposed to a subset of high-risk participants), RV does not pose a risk for these two autoimmune conditions in young children and is unlikely to be contributing to the increasing rates over time. This analysis, including more than two million participants, is the largest examination of RV and CD to date and the only examination of RV and AT to date.

Other studies report inconsistent findings as to whether vaccination against rotavirus may decrease risk of CD. Cohort studies of Rotarix vaccine® in the United Kingdom^21^ and RotaTeq in Finland^22^ did not find any association with rotavirus vaccination (RV). In contrast, one cohort of children with high-risk HLA haplotypes^23^ and another study of Finnish participants in a randomized controlled trial of RV reported decreased risk^24^. This lack of concordance between study results may be due to differences in the underlying populations studied and differences in ascertainment of vaccination status as well as CD, which was defined by a range of criteria including Scandinavian hospital-based registries^21,22^, biopsy^24^, antibody status^23^, or prescription of gluten-free goods^21^. Although data comparing the sensitivity and specificity of these criteria is lacking, data relying on provider diagnosis likely is less sensitive than cohort studies which actively monitor participants for antibody conversion or symptoms. The studies reporting associations performed more active surveillance in higher risk populations than studies not finding associations. It is also possible that children at higher risk due to HLA profile may derive greater benefit from RV due to higher incidence of CD, but that studies of populations with a wider range of baseline risk may not derive this benefit. Finally, it is possible that our report was underpowered, as the number of CD cases was low. However, using this database of over 2 million children, we have previously reported that rotavirus vaccination (RV) was associated with lower risk of type 1 diabetes^29^, similar to an ecologic studies from Australia^30^ and Israel^31^.

With respect to other risk factors for CD, our report was generally concordant with previous reports. We also found that children born prior to the introduction of RV had lower risk for CD compared to unvaccinated children born after the introduction of RV, consistent with reports that CD risk has increased over time^32^. Also consistent with prior reports were our findings of increased risk of CD among girls^21,33^; children residing in northern latitudes (particularly greater than 35°)^34^; and children with siblings with CD^35^. We found that greater comorbidity predicted greater incidence of CD. Although mechanisms are speculative, there are several possible explanations. Greater disease burden may increase vulnerability for CD through alteration in the microbiome or altered gut permeability and inflammation. Alternatively, slowed growth and development may be the initial presentation of CD, which has an insidious onset. Finally, children with a heavier disease burden may have greater contact with the health system and thus be more likely to be diagnosed. Of note, previous examinations of associations between CD and autism^36,37^ have noted significant associations potentially due to shared genetic vulnerability^38^ although the directionality of the association remains uncertain.

No other reports have examined associations between RV and AT. Despite sharing HLA alleles for susceptibility with CD and type 1 diabetes, neither Hashimoto’s disease or Grave’s disease are known to be linked with gastrointestinal infections to the extent of CD or type 1 diabetes. With respect to other risk factors for AT, our report was generally concordant with previous reports in that we found increased risk of AT among girls^39^. Other reports have not examined the relationship between region of the country and Hashimoto’s or Graves disease, or between comorbid conditions and risk of AT. Dietary iodine intake may be regional and also influences risk of thyroid disorders, although iodine deficiency is fairly uncommon within the United States. Of note, residence in New York or Pennsylvania was associated with increased risk for AT as well as CD and may reflect upstream factors including HLA susceptibility of the population in this region of the country and/or socioeconomic factors influencing access to care and other confounders.

Strengths of this report include the large size of the database, our ability to conduct comparisons of disease risk before and after introduction of the RV which reduces confounding associated with vaccination, examination of a broad population of risk, and capture of partial and incomplete vaccination status which does not reply on parental report. Our report has several limitations. First, due to the younger age of most of the children and the length of enrollment, we may not have captured associations between RV and onset of disease in slightly older children. As the majority of children in our study were enrolled for less than 7 years, it is possible that associations may have emerged with longer follow-up. This may be a limitation particularly for AT, for which the age of onset may be at slightly older ages than those observed for CD and T1D. Second, we relied on administrative claims data, which in turn relies upon physician diagnosis which may not entail biopsies or antibody ascertainment, and thus is likely less specific than studies incorporating these measures. In our examination, the prevalence of CD was 0.06%, compared with approximately 1% prevalence at the age of 7 years in population-based studies which screen all children for CD^3–5^. This disparity likely reflects the young age of our patient population since CD prevalence increases with age. It also reflects the fact that CD is underdiagnosed generally: in population-based studies, which perform serologies on all children, 90% of children with positive serologies do not carry a diagnosis of CD^1,2^. This disparity between diagnosed and undiagnosed CD may be due to lack of patient and provider awareness of the symptoms of CD and non-specific presentations: in general, symptoms are not highly correlated with transglutaminase autoantibody positivity^3^. Therefore, the lack of association between RV and CD in our study may reflect lack of association between the subset of children diagnosed with CD, rather than all children affected with CD. Among adults, persons more likely to have diagnosed CD as opposed to undiagnosed CD tended to have more comorbid conditions and to have greater healthcare utilization^5^. If this is also true in children, such patterns may have overestimated associations between RV and CD in children, and obscured any protective effect of RV. Along similar lines, the lack of association between RV and AT likely reflects lack of associations between the subset of children diagnosed with AT, rather than all children, although underdiagnosis of AT children has not been studied as extensively as underdiagnosis of CD. Third, we were unable to adjust for socioeconomic status, although we were able to adjust for regional variations in vaccination and CD which we have previously reported and thus reduced confounding by these variables and associated factors. Finally, it is possible that an interaction between vaccination and HLA status exists, where higher or lower benefit is observed among children at higher genetic risk. To our knowledge, such data examining HLA status as well as vaccination status is not available.

## Conclusions

We conclude that RV may not lead to reduced risk of CD and AT, even though it remains effective for its original intention or reducing risk of morbid gastrointestinal disease. Importantly, we did not find any increased risk with this vaccine, even accounting for several measures of childhood comorbidity. Thus, parents hesitant to vaccinate their children due to poor health status should be reassured that the risk of chronic conditions is minimal with this vaccine, and parents hesitant to vaccinate due to another child affected with autoimmune disease or autism should also be reassured that the risk of CD and AT with RV does not appear to be significant.

## Methods

We conducted a retrospective cohort study using de-identified claims data from Optum Clinformatics^®^ Data Mart, a product of OptumInsight, Inc. (Eden Prairie, MN). The administrative claims dataset includes more than 30 million commercially insured individuals each year from a geographically diverse US population (16% West, 20% Midwest, 36% South, and 27% Northeast) beginning January 1, 2001 and continuing to December 31, 2020^29^. These claim files include both commercially insured and Medicare Advantage enrollees, and covers the full set of service utilization such as inpatient hospitalizations, emergency department visits, other outpatient services, pharmacy, and laboratory claims. All enrolled beneficiaries must maintain both medical and pharmacy coverage throughout their insurance enrollment. Methods were carried out in accordance with relevant guidelines and regulations, and protocols were approved by the University of Michigan Institutional Review Board; informed consent was waived and approved by the University of Michigan Institutional Review Board.

We included children who had both medical and pharmacy coverage and who were born between January 1, 2001 and June 30, 2018 who were continuously enrolled in the health plan for at least 365 days, in order to ensure that we captured vaccination status. RV was classified in the mutually exclusive categories of no RV, born between 2001 and 2005; no RV, born during or after 2006; partial RV defined as ≤ 2 doses of RotaTeq or ≤ 1 dose of Rotarix; or the completed series defined as 3 doses of RotaTeq or 2 doses of Rotarix, prior to 8 months of age. Rotarix is an oral live attenuated vaccination derived from a strain isolated from calves. Rotarix has been proven to reduce incidence and severity of rotavirus gastroenteritis^40^. In a randomized trial of Rotarix, infants who received Rotarix had fewer rotavirus gastroenteritis episodes and fewer severe episodes caused by wild-type rotavirus compared to placebo, and only 5 infants had rotavirus gastroenteritis due to the vaccine, none of which were severe. Rotateq is also an oral live vaccination but derived from a strain isolated from a human. Rotateq has also demonstrated to reduce both incident gastroenteritis as well as severe episodes^41^. CD was determined using a claims-based algorithm and defined as ICD9/10 diagnosis codes 579.0/K90 and AT was defined as 245, E06.3, 242, 376.21, 376.22, E05.0, 579.0, K90 (codes for Hashimoto’s thyroiditis and Grave’s disease) at least a month apart after 1 year of age.

For examination of the association between RV and CD, Cox proportional hazards regression was used to compare time to CD in children by category of RV. In our base model, we adjusted for sex, birth year, region of country, and conditions which might confound decisions to vaccinate, namely history of allergy to vaccines and seizure history; and healthcare utilization as indicated by attendance of a well-child visit (Model 1). We conducted several sensitivity analyses. First, to determine if childhood frailty might be a confounder, an additional model adjusted for pre-term birth and childhood comorbidity as fixed covariates (Model 2). We used the modified claims-based version of the childhood chronic conditions score^42^, which incorporates neuromuscular, cardiovascular, respiratory, renal, gastrointestinal, hematologic or immunologic, metabolic, other congenital or genetic defects, and malignant neoplasms) between birth and 2 years. A point is assigned if a disease is present in each category, with the minimum score of 0 and a maximum score of 9; a higher score indicates higher comorbidity burden. Second, since parents might be less likely to vaccinate if they already had another child with CD, we also adjusted for older sibling history of CD using the approach described by Jain et al.^43^ (Model 3) Older siblings needed to also have been continuously enrolled for at least 6 months between the beginning and end of the study period (January 1, 2001 through December 31, 2019). Older siblings of index children were identified using a family identifier variable associated with the insurance policy; siblings had to be between 6 months and 17 years older than the index child to be included. Finally, we also adjusted for older sibling history of autism; although the association between vaccination and autism has been debunked, it is possible that this influenced parental decisions to vaccinate^43^. For examination of the association between RV and AT, similar models were constructed, except older sibling history of AT was substituted for older sibling history of CD. Seizure history and allergy to vaccination were modelled as monthly time-varying covariates.

Statistical significance testing of unadjusted rate ratios was conducted using the Yates X2 test, and statistical significance testing of hazard ratios estimated by maximum likelihood were conducted using Wald X2 statistics. Likelihood test ratios were used to test the statistical significance of Cox proportional hazards models with and without interaction terms. Proportional hazards assumption was assessed using the log rank test. All adjusted hazard ratios were estimated by maximum likelihood. All statistical tests were 2-sided and the alpha for all tests was 0.05.

We conducted several sensitivity analyses. First, we excluded children who had not attended a well-child visit. Children more likely to attend a well-child visit may have had more opportunity to receive vaccination, and parents who brought their children their children to such a visit may have been more attentive to symptoms consistent with autoimmune disease than parents who had not brought their children to such visits. Despite adjusting for well-child attendance in the main models, residual confounding was possible. Second, along similar lines, we excluded children who were born prematurely, since such babies are more vulnerable to infection and their parents might be more likely to vaccinate them and to monitor their health, including for symptoms of autoimmune disease. Third, we conducted analyses which examined whether the two RVs may have had separate effects by including an indicator variable for vaccination type, then examining the significance of the interaction term between the indicator and receipt of vaccination at p < 0.10. Fourth, we examined only children who were enrolled for more than 5 years, to address whether prevalence increased in this subset and also to determine if associations between RV and autoimmune disease were altered. Analyses were performed using SAS version 9.4 (SAS Institute Inc, https://support.sas.com/software/94/).

## Supplementary Information

Supplementary Tables.

## Data Availability

The data that support the findings of this study are available from Optum Inc., but restrictions apply to the availability of these data, which were used under license for the current study, and so are not publicly available. Data are available however with permission of Optum Inc.
