# Supplementary material for: Rotavirus vaccination is not associated with incident celiac disease or autoimmune thyroid disease in a national cohort of privately insured children
Source: Sci Rep. 2022 Jul 28;12:12941. doi: 10.1038/s41598-022-17187-y (PMC9334581; doi:10.1038/s41598-022-17187-y)
Supplement: Supplementary file 1 — Supplementary Tables. [file 41598_2022_17187_MOESM1_ESM.docx]

| Ancillary Table 1. Risk of celiac disease by rotavirus vaccination (RV) status. Association between other variables in models aside from RV with celiac disease. | | |
| --- | --- | --- |
|  |  |  |
|  | Hazard Ratio  (95% confidence interval) | p-value |
|  |  |  |
| Model 1, Adjusted for sex, year of birth, geographic region, vaccine allergy, history of seizures, and attendance of well-child visit |  |  |
| Female sex compared to male sex | 1.68 (1.51, 1.87) | <0.0001 |
| Year of birth | 0.98 (0.96, 1.01) | 0.15 |
| Geographic region (compared to Pacific region) |  |  |
| 0 (New England) | 1.95 (1.53, 2.49) | <0.0001 |
| 1 (New York, Pennsylvania) | 2.00 (1.55, 2.58) | <0.0001 |
| 2 (Mid-Atlantic) | 0.89 (0.69, 1.15) | 0.36 |
| 3 (Southeast) | 0.70 (0.55, 0.88) | 0.003 |
| 4 (Midwest) | 1.10 (0.87, 1.41) | 0.43 |
| 5 (North Central) | 0.93 (0.73, 1.18) | 0.43 |
| 6 (Mid Central) | 0.87 (0.68, 1.13) | 0.29 |
| 7 (South Central) | 0.77 (0.61, 0.97) | 0.028 |
| 8 (Mountain) | 1.51 (1.21, 1.88) | 0.0003 |
| 9 (Pacific) | Reference |  |
| Seizure history | 1.41 (1.10, 1.81) | 0.006 |
| Vaccine allergy | 1.80 (1.14, 2.83) | 0.011 |
| Well-child visit | 54.8 (7.74, 387.7) | <0.0001 |
|  |  |  |
| Model 2, Model 1 factors, and childhood illness |  |  |
| Preterm birth | 1.13 (0.94, 1.36) | 0.18 |
| Modified childhood chronic conditions score | 1.28 (1.26, 1.30) | <0.0001 |
|  |  |  |
| Model 3, Model 1 factors, and older sibling factors |  |  |
| Older sibling with CD | 68.3 (53.2, 87.7) | <0.0001 |
| Older sibling with autism | 1.88 (1.11, 3.19) | 0.019 |

| Ancillary Table 2. Risk of autoimmune thyroiditis by rotavirus vaccination (RV) status. Association between other variables in models aside from RV with autoimmune thyroiditis. | | |
| --- | --- | --- |
|  |  |  |
|  | Hazard Ratio  (95% confidence interval) | p-value |
|  |  |  |
| Model 1, Adjusted for sex, year of birth, geographic region, vaccine allergy, history of seizures, and attendance of well-child visit |  |  |
| Female sex compared to male sex | 2.31 (1.91, 2.80) | <0.0001 |
| Year of birth | 1.02 (0.97, 1.06) | 0.47 |
| Geographic region (compared to Pacific region) |  |  |
| 0 (New England) | 1.47 (0.94, 2.29) | 0.089 |
| 1 (New York, Pennsylvania) | 2.49 (1.65, 3.74) | <0.0001 |
| 2 (Mid-Atlantic) | 0.89 (0.69, 1.15) | 0.36 |
| 3 (Southeast) | 1.27 (0.89, 1.83) | 0.19 |
| 4 (Midwest) | 0.95 (0.65, 1.51) | 0.95 |
| 5 (North Central) | 0.63 (0.40, 0.98) | 0.04 |
| 6 (Mid Central) | 0.97 (0.64, 1.48) | 0.90 |
| 7 (South Central) | 0.89 (0.60, 1.30) | 0.53 |
| 8 (Mountain) | 0.58 (0.36, 0.93) | 0.023 |
| 9 (Pacific) | Reference |  |
| Seizure history | 2.80 (2.06, 3.81) | <0.0001 |
| Vaccine allergy | 1.64 (0.73, 3.68) | 0.23 |
| Well-child visit | (no convergence) | 0.93 |
|  |  |  |
| Model 2, Model 1 factors, and childhood illness |  |  |
| Preterm birth | 1.01 (0.77, 1.33) | 0.95 |
| Modified childhood chronic conditions score | 1.39 (1.35, 1.42) | <0.0001 |
|  |  |  |
| Model 3, Model 1 factors, and older sibling factors |  |  |
| Older sibling with AT | 38.7 (21.8, 68.9) | <0.0001 |
| Older sibling with autism | 1.84 (0.76, 4.44) | 0.18 |

| Ancillary Table 3. Sensitivity analyses excluding children who did not attend well-child visits, and sensitivity analyses excluding children who did not have pre-term births. | | |
| --- | --- | --- |
| Sensitivity analyses excluding children who did not attend **well-child visits** (n=1,821,683 children included in models) | Hazard Ratio (95% CI) | p-value |
| **Risk of celiac disease by RV status** |  |  |
| Adjusted for sex, year of birth, geographic region, vaccine allergy, history of seizures, preterm birth, childhood comorbidity score, older sibling celiac disease |  |  |
| No RV, born prior to introduction of RV | 0.69 (0.57, 0.83) | <.0001 |
| No RV, born after introduction of RV | Reference |  |
| Partial RV | 0.89 (0.72, 1.1) | 0.27 |
| Complete RV | 1.01 (0.86, 1.18) | 0.95 |
|  |  |  |
| **Risk of autoimmune thyroid disease by RV status** |  |  |
| Adjusted for sex, year of birth, geographic region, vaccine allergy, history of seizures, preterm birth, childhood comorbidity score, older sibling thyroid disease |  |  |
| No RV, born prior to introduction of RV | 0.8 (0.58, 1.09) | 0.16 |
| No RV, born after introduction of RV | Reference |  |
| Partial RV | 0.91 (0.63, 1.31) | 0.61 |
| Complete RV | 0.99 (0.75, 1.3) | 0.93 |
|  |  |  |
| Sensitivity analyses excluding children with **preterm births** (n=1,767,696) | Hazard Ratio (95% CI) | p-value |
| **Risk of celiac disease by RV status** |  |  |
| Adjusted for sex, year of birth, geographic region, vaccine allergy, history of seizures, well-child visit, childhood comorbidity score, older sibling celiac disease |  |  |
| No RV, born prior to introduction of RV | 0.68 (0.56, 0.83) | 0.0001 |
| No RV, born after introduction of RV | Reference |  |
| Partial RV | 0.89 (0.71, 1.12) | 0.33 |
| Complete RV | 1.06 (0.90, 1.25) | 0.47 |
| Risk of **autoimmune thyroid disease** by RV status |  |  |
| Adjusted for sex, year of birth, geographic region, vaccine allergy, history of seizures, well-child visit, childhood comorbidity score, older sibling thyroid disease |  |  |
| No RV, born prior to introduction of RV | 0.86 (0.61, 1.22) | 0.41 |
| No RV, born after introduction of RV | Reference |  |
| Partial RV | 1.02 (0.68, 1.53) | 0.91 |
| Complete RV | 1.15 (0.85, 1.54) | 0.37 |

| Ancillary Table 4. Sensitivity analysis examining only children who were enrolled for at least 5 years and thus were at least 5 years of age at end of enrollment (n=568,857). | | |
| --- | --- | --- |
|  | Hazard Ratio (95% CI) | p-value |
|  |  |  |
| **Risk of celiac disease by RV status** |  |  |
| Adjusted for sex, year of birth, geographic region, vaccine allergy, history of seizures, well-child visit, preterm birth, childhood comorbidity score, older sibling celiac disease |  |  |
| No RV, born prior to introduction of RV | 0.83 (0.65, 1.05) | 0.12 |
| No RV, born after introduction of RV | Reference |  |
| Partial RV | 1.01 (0.77, 1.32) | 0.96 |
| Complete RV | 1.09 (0.89, 1.33) | 0.41 |
|  |  |  |
| **Risk of autoimmune thyroid disease by RV status** |  |  |
| Adjusted for sex, year of birth, geographic region, vaccine allergy, history of seizures, well-child visit, preterm birth, childhood comorbidity score, older sibling thyroid disease |  |  |
| No RV, born prior to introduction of RV | 0.93 (0.67, 1.31) | 0.69 |
| No RV, born after introduction of RV | Reference |  |
| Partial RV | 0.8 (0.54, 1.2) | 0.28 |
| Complete RV | 0.92 (0.69, 1.23) | 0.57 |
